# Positioning public nursing colleges in South African higher education: Stakeholders’ perspectives

**DOI:** 10.4102/curationis.v42i1.1885

**Published:** 2019-05-23

**Authors:** Zanele P. Zwane, Ntombifikile G. Mtshali

**Affiliations:** 1 School of Nursing and Public Health, University of KwaZulu-Natal, Durban, South Africa

**Keywords:** nursing education, public nursing colleges, higher education, nursing profession, process of integration, university

## Abstract

**Background:**

Public nursing colleges (PNCs) are currently redeploying from provincial departments of health to higher education to become part of a unified higher education system in South Africa. As primary producers of nurses, this migration process needs to be managed carefully, with stakeholders having a common understanding of this process.

**Objectives:**

This study aimed to explore the stakeholders’ perspectives on the positioning of PNCs in higher education.

**Method:**

The study followed a qualitative grounded theory design. Purposive and theoretical sampling were utilised to achieve a sample size of 40 participants, including representatives from the Department of Higher Education and Training; professional associates; nursing educators; student leaders; nursing leaders; and nurses from the healthcare setting. Data were collected through observations, interviews and document analysis.

**Results:**

It emerged from the study that the integration of PNCs into higher education is a result of the country’s political and legal context. A number of policy and legal frameworks emerged as contextual conditions that provided a basis for the change. The integration of PNCs into higher education was conceptualised as a functional shift in the governance of colleges; a political tool to transform nursing education; a means to enhance the quality of college-based nursing programmes, and a vehicle for the greater professionalisation of nursing. Conflicting legislation and funding emerged as two issues of concern.

**Conclusion:**

Integrating PNCs with higher education came about because of political changes and the resolution of the ruling party to improve the quality of graduates produced, who will in turn improve the quality of healthcare service delivery offered.

## Introduction

Globally nursing education is undergoing reforms to strengthen the nursing and midwifery workforce, to enhance the professional status of nurses and to improve the performance of the healthcare systems in order to advance population health outcomes (World Health Organization [WHO] 2013, 2016). One of the major reforms is that of the integration of nursing education with the Ministry of Higher Education and Training as part of a unified higher education system (Blaauw, Dipotlo & Rispel 2014; Collins and Hewer 2014; Davies 2008; Dlamini 2011; Gobbi 2011; Mulaudzi, Daniels & Direko 2012; Muraraneza 2013; Spitzer & Perrnoud 2006).

Higher education is viewed as:

a vehicle for intellectual development, developing a flexible mind and regardless of the field of study, helping students acquire knowledge and intellectual skills that can be applied in a variety of different contexts. (Berret 2015:1)

This is lacking in most nursing graduates however, because of the technical focus of the education received on developing task-oriented skills, with limited development of intellectual, research and transferable life skills (Muraraneza & Mtshali 2018).

The integration of nursing education into higher education is in line with the recommendations by bodies such as the International Labour Organization (1976), the WHO (2009) and the International Council of Nurses (1986, 1997) that, where possible, basic nursing education should take place within the mainstream of the education system of a country. Countries such as the UK, Australia, Canada, USA, Denmark, Sweden, Finland, Norway, France and Switzerland underwent the process of integrating nursing education with higher education some years ago (Debout et al. 2012; Fletcher 2003; Råholm et al. 2010). Recently countries such as Swaziland, Rwanda, Nigeria and Lesotho have also reported adopting this reform (Agbedia 2012; Botma 2014; Dlamini 2011; Harerimana et al. 2015; Muraraneza 2013).

The positioning of public nursing colleges (PNCs) within higher education is differently defined by some, however, who view it as the migration, relocation, integration or incorporation of colleges to higher education or universities (Davids 2016; Department of Higher Education and Training [DHET] 2015; Matlakala 2016) as part of a unified post-school education and training system, as stipulated in the South African government’s 2013 *White Paper for Post-School Education and Training*. Others view this as a process of moving nursing education away from service settings or platforms to higher education settings to enhance the professionalisation of the field, as it is not a technical vocation or an on-the-job training qualification (Sowjanya & Subashini 2015). Yet others perceive the positioning of PNCs within higher education as a competency shift from the provincial Department of Health (DOH) to the national DHET in line with the prescripts of the Constitution of South Africa (*Act 108 of 1996*) and the *Higher Education Act of 1997*, as amended (Direko & Davhana-Maselesele 2017).

The history of formal nursing training in South Africa dates back to that provided by colonial missionaries in the 1880s, with the nurses undergoing on-the-job training that focused on the acquisition of technical skills (Dolamo & Olubiyi 2013). Later, nursing training became hospital-based under the country’s hospital administration. In the 1940s nursing education moved away from the hospital buildings to stand-alone nursing colleges, with buildings dedicated to training of nurses, under the provincial DOH, and this remains the case today (DOH 2012). According to Goodchild-Brown (1990), *Ordinance No. 12 of 1948*, amended by *Ordinance No. 13 of 1958*, and replaced in 1984, are the provincial ordinances that gave the colleges their legal status. The PNCs are, however, still associated with the hospitals they serve by acting as their clinical facilities.

Currently, nursing education in South Africa is provided using a variety of platforms including PNCs, universities and private nursing education institutions (NEIs). The 2014 South African Nursing Council (SANC) statistics showed that there were then about 135 active PNCs, including the 3 military schools, 101 private NEIs and 23 active university-based nursing departments, with one based in a private higher education institution (Van Rensburg 2015). Public nursing colleges remain the main producers of nurses (±80%) and are funded by the provincial DOH (DOH 2012; Mekwa 2000; Van Rensburg 2015). Unlike private NEIs and university-based nursing departments offering programmes accredited by both the SANC and Council on Higher Education, PNCs offer programmes that are only accredited by the SANC not the DHET (Uys, van Rooyen & Sheppard 2014; Van Rensburg 2015).

As part of legitimising the college-based nursing education, the van Wyk Commission, and also as the policy change made in 1986 by the SANC, mandated that PNCs enter into a form of affiliation with university-based nursing departments (Bezuidenhout 2014; Searle 1983). This was done to ensure quality in the programmes offered and to provide the required academic support (Blaauw et al. 2014; Dolamo & Olubiyi 2013; Mekwa 2000; South African Nursing Education Network [SANEN] 2014; Van Rensburg 2015). This type of support is still ongoing while working on the transitional arrangements to ensure that PNCs are positioned within the higher education system (Chief Nursing Officer, Nursing Policy Consultation Summit 2014). Post 2018, PNCs ‘may only provide higher education programmes under the authority of a higher education’ (DHET 2017), not in affiliation with the university-based nursing departments as has been the case since 1986, and they must apply for permission from the Minister of Higher Education to offer such programmes (DHET 2017).

The training of nurses in PNCs under the provincial DOHs has had a number of positive outcomes. These include the production of large numbers of nurses and midwives, which is in line with health service delivery needs, easy access to hospital settings for the immediate application of theory to practice and longer exposure to clinical learning because of the close proximity of hospitals used for experiential learning (DOH 2012; Van Rensburg 2015).

Contrarily, there is a widely held view that higher education equals better education for nurses, with graduates more competent and better equipped to deal with the complex challenges occurring within a dynamic healthcare system. In addition, they are equipped to cope with the increasing complexity of nursing practice, advances in medical and information technologies, and the demands of the ever-changing burden of diseases (Blaauw et al. 2012; Davies 2008; WHO 2009). Furthermore, nursing in higher education is associated with production of graduates with a broad knowledge base, an ability to conduct practice-related research and evidence-based practice abilities (Crenshaw & Champion 2013; Liao 2016; Roets, Botma & Globler 2016).

Countries that have undergone this change of mode to higher education caution that the process needs to be cautiously managed because of the challenges experienced and lessons learned when this was not carefully handled (Collins & Hewer 2014; Elkan & Robinson 1995). Some of the challenges associated with the abrupt move to higher education include the lack of a policy framework to guide the transitioning process; poor collaborative planning, unclear transitional arrangements; and the poor preparation of nurses and midwives for the changeover as both academics and clinicians. Authors such as Fealy (2004), Sliney and Uwimana (2014), Dlamini et al. (2014), Burke (2005) and Debell and Brandson (2009) highlighted these challenges, such as a reduction in the output of nurses, resulting in countries sourcing nurses from other countries; the drop in the competency levels of nurses as the university-based model moved students away from hospital training platforms (Dlamini et al. 2014, Fealy 2004), which allowed for the immediate theory-practice integration; and the disjuncture between academics’ and clinical professionals’ expectations from students. Hence, there is a need to have a clear understanding of what the process of moving PNCs to higher education means and what is entailed, in order to avoid those problems experienced by other countries.

### Problem statement

Legal prescripts in the country stipulate that education and training are a national competence under the DHET, and all post-school education programmes should be part of a unified higher education and training system. However, this is not the case with PNCs as they are still operating under the provincial DOHs and their programmes are accredited by the SANC not by the Council of Higher Education (CHE). Literature shows that the move to integrate PNC into higher education has taken a long time. This is associated with the complexity of the process of moving from policy to policy implementation (Blaauw et al. 2014); lack of direction regarding the exact positioning of nursing education (Matlakala 2016); and lack of clarity in the model to be adopted in integrating PNCs into higher education (SANEN 2014). Because of the urgency of integrating PNCs into higher education, authors such as Kraak (2012) and Uys et al. (2014) are of the view that whichever option is adopted, the priority remains the development of a coherent framework that will allow the PNCs to fit into the post-school system. The positioning of PNCs outside higher education, as well as the poorly defined process of integration into higher education, is causing confusion and uncertainties and is posing a significant risk to the maintenance of a coherent education system (Van Rensburg 2015). Hence there is a need to understand stakeholders’ perspectives on the phenomenon of positioning PNCs in higher education in order to inform the change process.

### Aim of this study

This study aimed to explore the nursing education stakeholders’ perspectives on the positioning of PNCs within South African higher education.

### Research question

How do nursing education stakeholders understand the phenomenon of positioning PNCs within higher education?

## Research method and design

Guided by the sociological–symbolic interactionist stance, this study followed a qualitative and grounded theory approach, which is commonly used in studying an unfamiliar phenomena (Corbin & Strauss 2008, 2015), although there are certain preconceptions and assumptions that exist about it (Maijala, Paavilainen & Astedt-Kurki 2003). Symbolic interactionism is premised on the assumption that individuals act towards things on the basis of the meanings they attach to those things, and as such the meaning emerges from the social interactions individuals have with their counterparts (Chamberlain-Salaun, Mills & Usher 2013). ‘The meanings are handled in, and modified through, an interpretive process used by the person in dealing with the things he [*sic*] encounters’ (Blumer in Chamberlain-Salaun, Mills & Usher 2013:5). Grounded theory was chosen because it provides a flexible and practical way of interpreting a complex and unfamiliar social phenomenon (Charmaz 2003), which in the context of the study was the phenomenon of integration of PNCs into higher education – a phenomenon about which little is known in South Africa and an area in which there is a dearth of literature.

### Setting description

In grounded theory, data is collected in natural settings because the context of the research setting is central to the meaning generated (Corbin & Strauss 2008, 2015; Lincoln & Guba 1985), as was the case for this study, in order to obtain rich data. In this particular study, the researcher selected two PNCs that were actively engaged and at an advanced stage in the process of preparing for integration into the higher education sector. These two settings were targeted to enhance the richness of the data collected and strengthen the emerging theory, which is grounded in diverse perspectives, as stated by Creswell (2007).

### Sampling procedure

Purposive and theoretical sampling were used to ensure that the selected participants had rich information to contribute to the study, as stipulated by Corbin and Strauss (2015). A total number of 40 respondents participated in this study. They included representatives from DHET (1); the Democratic Nursing Organisation of South Africa (DENOSA) (1); nurse leaders (4); principals (3); college registrars (2); nursing educators (15); clinical facilitators (5); and Student Representative Council (SRC) members (9).

### Data collection

The process of data collection and initial data analysis continued over a period of 2 years (2016–2018) until theoretical saturation, as stated in Corbin and Strauss (2015), was reached. Data were collected through observations, document analysis and individual and focus group interviews to gain multiple perspectives and thereby obtain rich data (Corbin & Strauss 2015). Observations and document analysis were performed concurrently before conducting interviews. Observations, guided by an ‘observation guide’, sought to elicit the magnitude and structural design of changes made in preparation for integration, as well as of teaching and learning environment enhancement initiatives. Document analysis was conducted in preparation for the focus group interviews. The document analysis tool used focused on the key issues related to the integration process. All available documentation produced after the initiation of the integration process was utilised.

Documents included minutes of the meetings held, curriculum development committee minutes and other related reports and documents, including legal and policy frameworks, which were used as references. In-depth interviews were used to collect data from the representatives from DHET and DENOSA, the nursing manager, senior nursing leaders, college principals and registrars, clinical facilitators and the nine other nursing educators who were members of curriculum development teams.

Data from six nursing educators and the SRC were collected through focus group interviews. The focus groups were limited to a maximum of six members to encourage active participation. The interviews were used to clarify, verify or confirm information obtained through observations and document analysis. Each focus group interview lasted from 50 min to an hour, while individual interviews lasted for 45–50 min. The interview guide consisted of questions such as ‘Please explain how you understand the integration of PNCs programmes into higher education’, ‘What has led to this need to integrate nursing education programmes into higher education?’ and ‘What is the rationale for this change?’, with probes such as ‘Is there any policy document that states that PNCs have to offer higher education qualifications?’ and ‘What do you see as possible outcomes of this process of integration of PNCs’ programme into higher education?’. Both focus group participants and individual interviewees responded to the same research questions. Interviews were audio-taped, with permission from the participants. Copious field notes were taken during the data collection process to obtain rich data and ensure that in-depth descriptions were provided (Charmaz 2003). Using memos, the researcher kept a record of information concerning the research process as it unfolded, the emerging perspectives from the data and the study’s substantive findings (Corbin & Strauss 2015).

### Data analysis

The QSR NVivo version 10 software was used to organise the data. The constant comparative method of data analysis was applied, constantly comparing emerging data codes to codes, codes to categories, categories to categories and continually reassessing meanings in order to understand what was going on from the perspective of the participants (Corbin & Strauss 2015). The three phases of data analysis proposed by Strauss and Corbin (1990) – open coding, axial coding and selective coding – were followed. During open coding the researcher, through line-by-line analysis, discovered and identified concepts and their properties from the data. This phase entailed breaking down, examining, comparing, conceptualising and grouping data with the same meaning into categories (Corbin & Strauss 2015; Strauss & Corbin 1990). During axial coding, the researcher described the concepts in terms of properties and dimensions and used a coding paradigm to connect categories found during open coding. The process of connecting related categories into single categories assisted in reducing the number of initial categories by ensuring that they were organised in a meaningful and hierarchical manner (Corbin & Strauss 2015). Selective coding was the final phase, which occurred over time. It entailed integrating, unifying and refining the emerging theory, as well as verifying and validating categories against the data and selected literature. The core category and supporting categories were clearly captured, showing relationships between and amongst concepts, depicting the emerging storyline (Corbin & Strauss 2015) of stakeholders’ perspectives and revealing an understanding of the phenomenon of positioning PNCs within the South African higher education system.

### Trustworthiness

Lincoln and Guba’s (1985) methods of trustworthiness – credibility, dependability, transferability and confirmability – were adopted in order to enhance the quality and reliability of the study findings. The emerging findings were double-checked with the participants and against the data, and a co-coder was used to confirm the emerging codes and categories for credibility. Dependability was ensured by triangulating data sources, validating transcribed interviews with the participants and double-checking the emerging codes and categories with the research supervisor. Providing thick descriptions of the study context, settings, procedures and findings assisted in ensuring transferability. Confirmability was ensured by matching collected data against the participants’ original understanding of the phenomena of interest in this study.

### Ethical considerations

Ethical approval was obtained from the University Research Ethics Committee and gatekeeper permission was sought from the Provincial Departments of Health and principals of the two colleges of nursing. Informed consent was obtained from all participants before data collection and ethical principles were adhered to, as was stated in the research protocol. Ethical approval for this study was obtained from the University Research Ethics Committee (protocol reference number: HSS/0351/016D), Mpumalanga Provincial Health Research and Ethics Committee (PHREC REF:MP-2016PR14-195) and KwaZulu-Natal Provincial Health Research and Ethics Committee (HRM130/16 KZ 2016 RP32-809).

## Results

Four categories were generated in this study: (1) antecedents to the positioning of PNCs in higher education, (2) contextual conditions, (3) conceptualisation of positioning PNCs within higher education and (4) anxieties related to the integration of PNCs into higher education.

### Antecedents to the positioning of public nursing colleges in higher education

Antecedents are those events or incidents that occur prior to or lead to the occurrence of a phenomenon (Walker & Avant 2005), which in this study is the integration of PNCs into higher education. Three domains emerged under the antecedent conditions category: (1) political changes in the country, (2) political resolution of the ruling party and (3) global reforms in nursing education.

Political changes in the country: It emerged from the study’s data sources that nursing education prior to democracy in South Africa was fragmented and characterised by segregation, inequality and inconsistency. It was also discovered that this picture was not unique to nursing education but was likewise observable in other sectors in the country, leading to the implementation of the national Reconstruction and Development Programme (RDP).

According to the study respondents, issues relating to nursing education were partially addressed through the RDP, with PNCs undergoing rationalisation from approximately 2005. It is reported that these colleges underwent mergers to reduce the number of existing institutions and streamline them under one governance structure; use a common curriculum; and adopt common teaching and learning policies. This change was not enough, however, according to study respondents, as PNCs continued to offer nursing education programmes under the provincial DOH, outside of the higher education platform, which falls under the jurisdiction of the Minister of Higher Education.

‘Post 1994 the country had an RDP plan … to correct the inequalities of the past, the fragmented and inequitable nursing education system in our context. Through this programme, in our province a single nursing college was born in 2005, which amalgamated 25 nursing education institutions throughout the province. We adopted a uniform curriculum, teaching and learning policies, and examination and student management system. This change was however not enough, as we remained isolated, outside the higher education system.’ (P05, female, nurse educator)

Political resolution of a ruling party: Fortifying the drive to move PNCs from provincial DOHs to higher education is the 2008 political resolution of the ruling party emanating from the Mangaung Conference. This political resolution stipulates that public colleges operating outside the Department of Higher Education under the provincial departments should be integrated into higher education as part of the mainstream higher education system. Public nursing colleges were one of the groups of the colleges focused on in this political resolution:

‘… the Mangaung ANC conference … 2008, resolution was that … nursing colleges should be transferred from the provinces … under the provincial departments … to the national DHET in accordance with the constitution.’ (P12, female, nurse educator)

Global reforms in nursing education: It emerged from this study that nursing education in South Africa is influenced by the global reforms in nursing education. One of the reforms pointed out by the respondents was the moving of nursing education to the tertiary or higher education system. According to respondents, South Africa was lagging behind, as some countries underwent this process more than 15–20 years ago, as per this response:

‘We do not have an option but to align ourselves as colleges with global trends in nursing education. SA is starting so late compared to other countries. Some countries have finished this process 15–20 years [*ago*] so we are very backwards in terms of the movement, but it is happening in South Africa.’ (P10, female, nurse educator)

### Contextual conditions

Contextual conditions are the structural provisions that serve as enablers, legally legitimising and supporting the integration process. Subcategories falling under this category include: (1) the DHET, the SANC and national DOH legislative frameworks and (2) the National Strategic Plan for Nursing Education, Training and Practice 2012/13–2016/17. These, according to study sources, provide the basis for positioning PNCs in higher education.

#### Department of Higher Education and Training, South African Nursing Council and National Department of Health legislative frameworks

The *Higher Education Act* 101 of 1997, as amended, emerged as a significant driver in the introduction of the PNC integration process. The study respondents highlight that this act is based on the *Constitution of the Republic of South Africa* No. 108 of 1996, which states that all tertiary education must be a national competence, thereby placing the sole responsibility of education and training on the DHET. According to respondents, the prescript of the *Higher Education Act* implies that nursing education should be offered under the higher education platform. The *Government Gazetted Act*, No. R. 801 of 2017, by the DHET on phasing out old nursing qualifications adds impetus to PNCs as per these respondents’ comments:

‘The *Higher Education Act* of 1999, which is based on the country’s constitution, which clearly stipulates that tertiary education is a national competence under the Minister of Higher Education.’ (P12, female, nurse educator)

The Government Gazette … of 2016 … stipulates December 2019 as a last date for the intake of all programmes leading … not complaint with … the *NQF Act*, provided an impetus to nursing colleges to seriously work towards the integration to higher education. (Document analysis)

Another driver, according to the data sources, was the *NQF Act* (Act No. 67 of 2008), which required all programmes to be aligned to this framework, including the nursing education programmes. Respondents explained that this act posed a potential challenge in nursing output by all nursing programmes, as nursing education qualifications are now pegged at the higher education band of the National Qualifications Framework (NQF), which requires nursing qualifications to be offered in higher education. Some of the comments from study respondents were that:

‘… the *NQF Act* of 2008 initiated transformation in the education system … so the PNCs had to be taken away from the provincial DOH to higher education to be integrated into the higher education prescript to continue to offer NQF-aligned qualifications.’ (P28, female, senior nursing leader)

‘… so automatically the public colleges, if they do not respond to the act, will have to fall off the wagon in terms of higher education prescript.’ (P14, male, senior nursing leader)

The *White Paper for Post-School Education and Training* (2013) emerged as another important legal framework that compels PNCs to comply by offering programmes that meet the higher education standards and that are offered within higher education as part of the post-school education and training system. As per one respondent:

‘… The *White Paper for Post-School Education and Training* stipulates that all post-school education [*is*] to be under the Minister of Higher Education and Training and that include nursing education in PNCs.’ (P07, female, student)

The SANC Nursing Education Qualifications framework, together with the regulations on nursing education and training programmes, emerged as important legal frameworks that made it mandatory for PNCs to revisit their nursing education programmes and ensure that they were aligned with them.

#### Strategic plan for nurse education, training and practice

Respondents referred to the Strategic Plan for Nurse Education, Training and Practice 2012/13–2016/17 as another legal document that necessitates the integration of PNCs into higher education and that sends a call for nursing education to be regarded as a national competence:

‘The nursing strategy calls for PNCs currently functioning under the provincial Departments of Health to become a national competence under the National Department of Health and offer programmes aligned to the requirements of higher education …’ (P14, male, senior nurse leader)

‘The strategy indicates that nursing education and training should be a national competence … one of the strategic objectives in the Nursing Strategy states that all NEIs should be positioned as higher education institutions.’ (P06, female, nursing manager)

### Conceptualisation of positioning public nursing colleges within higher education

A number of perspectives were generated with regard to understanding of the phenomenon of positioning PNCs within higher education. These were grouped into four subcategories: (1) a functional shift in the governance of PNCs; (2) a political tool to transform nursing education in public colleges; (3) a means to improve the quality of college-based nursing education programmes; and (4) a vehicle to allow for the greater professionalisation of nursing.

#### A functional shift in the governance of public nursing colleges

The integration of PNCs into higher education was, according to study respondents, perceived as a functional shift in their governance, of PNCs, from the provincial to the national level. According to respondents, in the past nursing was a provincial competence under the provincial DOHs and now, according to the country’s constitution, it is a national competence under the DHET. It emerged that DHET may delegate responsibility in this regard to the national DOH:

‘I view the move of PNCs from the provinces to the national level as a matter of governance shift in line with the country’s constitution and *Higher Education Act*. The training is becoming a national competence in terms of how it will be governed to ensure standardisation, uniformity across the country. DHET may delegate this responsibility to [*the*] national DOH.’ (P07, female, student)

‘… the act states that the Minister of Higher Education and Training may delegate the function of managing tertiary education to another state entity, in this particular case the national DOH …’ (P18, female, nurse educator)

It emerged from the respondents that the PNCs were not physically moving to higher education but would instead be governed by the legal and political frameworks of higher education. There was an understanding that PNCs would remain under the DOH but offer programmes that adhere to the accreditation standards of higher education and the regulatory frameworks and policies of the DHET, which was not the practice in the past. In addition, the nursing colleges will enter into a memorandum of understanding with the universities in their geographic areas, and not with university-based Departments of Nursing, which was also the case in the past:

‘We will be governed by the higher education legal frameworks but under the DOH … We are not moving physically to universities. The academic quality assurance will remain the primary function of the DHET.’ (P28, female, senior nursing leader)

‘… nursing colleges are not coming to higher education per se but will offer programmes accredited by DHET. They will have a memorandum of understanding with the university in the area, not the nursing department, which has been the practice since 1986 …’ (P01, female, college principal)

#### A political tool to transform nursing education in public colleges

Moving PNCs from the provincial DOH to higher education was perceived as an agenda of the democratic South African government in formulating a unified higher education system in the country, with all education programmes under the DHET, in line with the country’s constitution. This transformation, according to study respondents, will address the issue of a fragmented nursing education system, which is characterised by inequities because of the legacy of the previous government:

‘Currently nursing education falls under the Department of Health … With the integration, nursing colleges will now fall under the Ministry of Higher Education so they will be under higher education.’ (P06, female, nursing manager)

‘Integration of PNCs to higher education … means bringing together nursing education into one national system of education to address the limitations of the nurse training before 1994 that mostly affected nurses produced from colleges.’ (P11, female, college principal)

Elaborating on addressing the legacy of the old government, the respondents also indicated that they saw the move to higher education as a solution for the long route to which college-trained professional nurses are subjected before they have access to postgraduate studies. The move will enable them to progress to postgraduate studies, much like their counterparts qualifying from university-based nursing departments, who are also registered with the SANC as professional nurses. Respondents reported that college-produced professional nurses perform a minimum of 7 years’ study before they qualify to access postgraduate studies, irrespective of their years of work experience and the post-basic qualifications they have obtained.

The old system favoured university-based graduates, which in the past was not accessible to other groupings in the country. This impedes the upward career movement of nursing trainees in these other groupings, as well as their personal development as professionals. With colleges offering NQF-aligned qualifications this legacy will be eliminated, as the framework clearly stipulates articulation possibilities for each qualification. Below are given supporting extracts:

‘One of the legacies of the old system is that professional nurses produced from the colleges are unable to progress to postgraduate programmes like their university-produced counterparts. It takes them 7 to 8 years … before qualifying to further their studies at universities. … This move will eliminate this bad legacy.’ (P11, female, college principal)

‘The system delayed the professional nurses produced from colleges to register for university-based postgraduate programmes. Unfortunately, college-based education was designed for students belonging to certain groupings and university education was for a limited few that belonged to a privileged group in the country. Nurses from colleges ended up moving from one post-basic course to another, with each programme existing in isolation, making them repeat the learning content. Now they will obtain qualifications aligned to the NQF, which stipulates progression and articulation routes.’ (P01, female, college principal)

Some respondents highlighted the issue of a student’s status in college as being a legacy of the past, with students having both an employee and a learner status. In their view the college-based learners will have a full student status, like the university-based students, with learning as a priority, to try and meet the conditions of the employers while fulfilling all student requirements. According to the following three study respondents, this will enable students to have greater educational freedom, which will allow them to prioritise learning and spend time in clinical settings developing the required competencies, as per the extracts below:

‘… Nursing students will have the status of being full students … just like all other higher education students, instead of the current dual status of an employee and student. They will have an opportunity to fully dedicate their time to learning and developing the required competencies like the university nursing students …’ (P12, female, nurse educator)

‘… This means change on the side of the students from the college … currently, there is over-reliance of the hospitals on their [*students’*] services as they are also employees, instead of creating learning opportunities and meeting the students’ learning needs … with service needs taking priority over learning needs.’ (P22, male, student)

‘… the students will have more time to focus on learning … developing the required competencies instead of task-oriented skills …’ (P02, female, nurse educator)

#### Means to enhance the quality of college-based nursing education programmes

Study participants viewed the process of integrating colleges and college-based nursing education programmes with universities as a means to enhance the quality of their education programmes. The PNCs and their programmes will, for the first time, be subjected to dual accreditation from the Higher Education Quality Council of the CHE and the SANC.

The rigorous accreditation process adopted by these two external bodies will, according to the study sources, enhance the quality of nursing education programmes offered in PNCs. Unlike other NEIs, the private and university-based nursing departments and programmes offered by PNCs are subject only to the SANC accreditation process, which will change their move to higher education, as per the following responses:

‘Academic quality assurance and the educational aspects surrounding the training of nurses will be done in collaboration with the DHET, the CHE and SANC …’ (P18, female, nurse educator)

‘… The Nursing Colleges will have to adhere to the 19 criteria of the CHE in that Programme Accreditation Criteria document of 2004 … They will undergo the dual accreditation process by SANC and the CHE and that will improve the quality of programmes offered by the colleges.’ (P12, female, nurse educator)

Respondents and empirical data revealed that PNCs are upgrading their teaching and learning infrastructures, libraries, classrooms, clinical skills laboratories and computer laboratories to ensure that these meet the higher education accreditation standards. They are also reformulating and adopting innovative curricula and new teaching, learning and assessment strategies in line with the national and provincial priorities. In addition, the PNCs are investing in building the capacity of nursing educators to meet the higher education system standards. The outcome of these interventions, according to respondents, is an improvement in the quality of nursing education programmes, resulting in better-prepared graduates:

‘ … it entailed infrastructure development, we now have like [*a*] functional computer lab, because higher education is going to need more research being done by students, upgraded library because students will be more actively involved in their learning, so they need to go and find information for themselves, and newly built clinical skills laboratory.’ (P10, female, nurse educator)

‘… upgraded the lecturers, because to teach in higher education you need to have at least the master’s: when the new qualification starts most of them will be, having the master’s.’ (P22, female, student)

#### A vehicle to allow for the greater professionalisation of nursing

Some participants were of the view that the move to higher education was one way of enhancing the professional status of nursing, by affording the nursing profession the same status as other professions, which will strengthen the capacity of nurses for autonomous research and evidence-based practice. Public nursing colleges were presented as the main producers of nurses and midwives, which was estimated to be at about 80%.

Although this is appreciated, the concern is that programmes at PNCs show limited focus on research and evidence-based practice, which is required to improve the quality of healthcare and health service delivery. The move to higher education, with programmes having adequate research content and application of research to practice, was positively perceived, in that the system will be injected with more nurses skilled in research and evidence-based practice. This will in turn improve the quality of graduates produced and enhance the professional status of nurses, with:

‘… nursing being treated as any other professions … It will improve the quality of nurses … who will be able to function independently in a system that is dependent on nurse … equipped for advances in medical and information technology because of the subjects they will be expected to take in addition to nursing courses …’ (P01, female, principal)

‘… Colleges will offer programmes with more focus on [*being*] research focused, preparing students to provide evidence-based care and to be able to function independently. This has been the gap from our colleges and a concern because the majority of nurses in the system, about 80%, come from us.’ (P07, female, student)

‘… offering nursing qualifications at the higher education level … like all other professions … will put nursing at the level where it is supposed to be as a profession.’ (P10, female, nurse educator)

### Anxiety related to the integration of public nursing colleges into higher education

Data from the study revealed two concerning issues about moving PNCs to higher education. These included (1) conflicting legislation and (2) the funding of PNCs.

#### Conflicting legislation

Study respondents pointed out three conflicting pieces of legislation in terms of those responsible for the education and training of nurses. These legislative frameworks include the *Higher Education Act*, the *National Health Act* and the *Nursing Act*. According to the *Higher Education Act*, which is premised on the constitution of the country, the DHET is responsible for all education and training post-school. The *Health Act*, on the other hand, stipulates that the national DOH under the Minister of Health is responsible for the training of human resources for health (HRH), with the *Nursing Act* indicating that the national DOH and the SANC are responsible for institutions that produce nurses. The understanding from one of the respondents was that this will be addressed through the new higher education bill, which will override these legislative frameworks, as per the following extract:

‘… It is a major problem … contradictions between three acts, the *Higher Education Act*, the *Nursing Act* and the *National Health Act* … They contradict each other in terms of who has to take the responsibility for what? … The *Higher Education Act* says that the DHET is responsible for all institutions of post-school education and training, but the *Health Act* says the National Department of Health is responsible for the training of HRH. The *Nursing Act* says the National Department of Health and the SANC are responsible for the institutions that produce nurses. So now, who is correct? And that is one of the things that is being sorted out by the new higher education bill, which I say will supersede the *Nursing Act* and the *Health Act*.’ (P12, female, nurse educator)

#### Funding issues

Funding of PNCs emerged as another area of concern, because colleges are currently fully funded by the provincial DOH, including student funding. The higher education system, with its funding framework, may not be able to cater for the large numbers of student nurses required by the DOH in line with its HRH plan. This, according to the study respondents, may compromise the supply of the number of nurses needed.

Research further revealed that the move might affect the funds allocated to provincial DOH for PNCs, as funded by the national DOH and the National Treasury. These funds may instead be allocated to the Health Department at a national level, which will then be mandated to take responsibility for PNCs, as per the following responses;

‘… there is a problem when it comes to funding of the nursing colleges … nursing colleges at the provincial level are the primary training space for nurses in accordance with the provincial Department of Health HRH plans. We train large numbers. Will DHET manage to fund all these student nurses with their funding framework? That is our concern, because if they don’t, we will have to train less and that will have impact on the supply that is in the HRH plan …’ (P25, female, clinical facilitator)

‘… the function shift has to go along with the movement of money from the treasury … the money will no longer go from the national Department of Health to the provinces, but it will go from the national treasury to the responsible branch of the national Department of Health or Higher Education … because you cannot shift a function and not shift the money. The money must follow the function.’ (P13, female, representative from DHET)

This study showed that integration of PNCs into higher education would increase the quality of the nursing workforce in their research skills and evidence-based practice. Lecturers should hence be provided with opportunities for capacity building in these areas of practice.

## Discussion

This study revealed that the dialogue on the integration of nursing education, especially PNCs, has been in existence for some time, as stated in Mekwa (2000), but is gaining momentum now because of the available political will, enabling legal frameworks and negative implications for the healthcare system if the colleges are not integrated to higher education. The 2008 Mangaung political resolutions set in motion the integration of public colleges from the provincial health departments to the DHET. In this study, the moving of these colleges was perceived as a political tool used by the governing party to address the inequalities of the past, which happen to have affected mainly the public colleges. Perceiving this reform in nursing education as the current government’s political tool to bring about change is in keeping with Paulo Freire’s theory that the education system can be used as a tool to achieve the government’s political agenda (Harris 2004). Nursing education can therefore not be isolated from the political reforms in the country.

The positioning of PNCs in higher education does not necessarily mean physically relocating PNCs, but it was understood in this study as being a shift in their governance function from the provincial departments to the national department in line with the country’s constitution, which holds education to be a national competence. Lindblad, Johannesson and Simola (2002) report a similar situation in the UK, where the change in this regard was mainly in the governance of NEIs. As stated by Van Rensburg (2015), it emerged that the Minister of Education might delegate the function to the national DOH to ensure that the production of nurses is not compromised.

With the decision to delegate in this way, National Department of Health (NDOH) takes into consideration the high cost and complexity of training large numbers of nurses to respond to the country’s health service delivery needs. Francis and Humphreys (1998) similarly report that financing of nursing education in the UK remained under the Ministry of Health. The high cost of training nurses in higher education was pointed out as the primary prohibiting factor for the integration of colleges of nursing into higher education (Collins & Hewer 2014; Spitzer & Perrenound 2007). According to these authors, keeping the funding with the Ministry of Health ensured that nursing education courses would primarily reflect the service needs, and the numbers admitted to the programme, as guided by the HRH training plans. Australia, however, reported that funding was transferred to the Department of Employment, Education and Training (Francis 1999).

A range of models for positioning nursing education in higher education exist, which include moving colleges to the academic tertiary level, to universities, to the vocational level, pairing them with universities for quality assurance purposes or declaring colleges to be autonomous institutions (SANEN 2014; Spitzer & Perrenoud 2007; Van Rensburg 2015; Zabalegui et al. 2006). The model for the positioning colleges in higher education that emerged from this study is that of having colleges enter into an agreement with universities, rather than being university-based nursing departments, as was the case in the past. Spitzer and Perrenoud (2007) reported that the French-speaking region in Switzerland positioned nursing education at the academic tertiary level, while German-, Italian- and Romance-speaking regions in the same country placed nursing programmes at the vocational rather than the academic tertiary level.

Integrating PNCs into higher education was associated with improved quality of patient healthcare and health service delivery in this study, in that graduates will be equipped with discipline-specific skills, as well as research and evidence-based practice skills, which are currently not the focus in programmes offered by PNCs. In this study, this was viewed as a step in the right direction, because a dynamic healthcare system characterised by complex cases and well-informed clients requires a broadly prepared nursing complement, with good research and problem-solving skills, accompanied by the ability to use the existing latest existing clinical evidence in their practice (Baloyi & Mtshali 2018; Tyrrell 1998). According to Aiken (2003), Collins and Hewer (2014) and Fitzpatrick, While and Roberts (1993), higher education plays a significant role in producing broadly prepared nurses possessing sound scientific grounding, which is essential to improving patient outcomes.

Integration of PNCs emerged as a vehicle for the greater professionalisation of nursing, as with other healthcare professions. Nursing is more than a vocation; it is a profession with its own body of knowledge. According to Barton (1998), the integration of nursing colleges with higher education provides joint validation, which allows for the awarding of both academically and professionally recognised qualifications. The struggle for professionalisation of nursing is associated with gender issues, as nursing is historically a female-dominated profession working closely with other male-dominated professions. This pattern is observed globally.

This study revealed that the process of integrating PNCs into higher education needs to be carefully managed, with clear policies and legal frameworks implemented to avoid the issues that were reported in other countries with regards to moving nursing to higher education. Two issues emerged in this study that require urgent attention: the conflicting policy and legal frameworks, and funding. Issues reported in other studies include a lack of clarity and consistency in terms of policy direction regarding transitioning to higher education (Burke 2005; Debell & Branson 2009); poor communication to stakeholders (Mcqueen & Grenier 1993; Reddy 2007); a drop in clinical and learning standards and clinical learning support to students (Dlamini 2011); and job losses as well as closure of those NEIs that did not meet the higher education accreditation standards (Gillet 2010). According to Buthelezi (2016), however, the success of educational reforms should be evaluated rather by the degree to which such changes are understood and fully adopted by their recipients.

## Recommendations

This study showed that the legislative framework regulating the education and training of nurses is characterised by contradictions. Moving forward, it is recommended that the ministers of the DOH and DHET communicate with stakeholders regarding the conflicting legislation to avoid confusion. There is a need for a joint communication from the DHET, the DOH and the SANC as governance structures to the stakeholders about the integration process with regard to what this process entails and also concerning stakeholder expectations.

## Limitations of the study

The researcher was unable to gain access to other critical stakeholders at national level. Gatekeeper permission was requested but could not be obtained, and theoretical sampling assisted in this regard. The researcher managed to collect data from independent nursing leaders at national level who were recommended by study participants.

## Conclusion

The study data revealed that the positioning of colleges of nursing within higher education came about because of antecedents, namely political changes in the country; the political resolution of the ruling party; and global reforms in nursing education. What set the integration process in motion was the DHET, DOH and SANC legal frameworks and strategic plan for nursing education, training and practice. According to the study results, respondents viewed the integration of PNCs into higher education as a functional shift in the governance of these colleges; a political tool to transform nursing education in public colleges; a means to improve the quality of college-based nursing education programmes; and a vehicle for the greater professionalisation of nursing.
